# Intra- and extra-cranial effects of transient blood pressure changes on brain near-infrared spectroscopy (NIRS) measurements

**DOI:** 10.1016/j.jneumeth.2011.02.029

**Published:** 2011-04-30

**Authors:** Ludovico Minati, Inge U. Kress, Elisa Visani, Nick Medford, Hugo D. Critchley

**Affiliations:** a Department of Psychiatry, Brighton & Sussex Medical School (BSMS), Falmer, UK; b Scientific Department, Fondazione IRCCS Istituto Neurologico Carlo Besta, Milano, Italy; c Department of Neurophysiology, Fondazione IRCCS Istituto Neurologico Carlo Besta, Milano, Italy; d Sussex Partnership NHS Foundation Trust, Brighton, UK; e Sackler Centre for Consciousness Science, University of Sussex, Falmer, UK

**Keywords:** Near-infrared spectroscopy (NIRS), Blood pressure, Autonomic arousal, Visual stimulation

## Abstract

Brain near-infrared spectroscopy (NIRS) is an emerging neurophysiological tool that combines straightforward activity localization with cost–economy, portability and patient compatibility. NIRS is proving its empirical utility across specific cognitive and emotional paradigms. However, a potential limitation is that it is not only sensitive to haemodynamic changes taking place in the cortex, and task-related cardiovascular responses expressed in the perfusion of extracranial layers may be confounding. Existing literature reports correlations between brain NIRS and systemic blood pressure, yet it falls short of establishing whether in normal participants the blood pressure changes encountered in experimental settings can have confounding effects. Here, we tested this hypothesis by performing two experimental manipulations while recording from superficial occipital cortex, encompassing striate and extrastriate regions. Visual stimulation with reversing chequerboards evoked cortical haemodynamic responses. Simultaneously and independently, transient systemic blood pressure changes were generated through rapid arm-raising. Shallow-penetration NIRS recordings, probing only extra-cerebral tissues, highlighted close haemodynamic coupling with blood pressure. A different coupling pattern was observed in deep-penetration recordings directed at haemodynamic signals from visual cortex. In absence of blood-pressure changes, NIRS signals tracked differences in visual stimulus duration. However when blood pressure was actively manipulated, this effect was absent and replaced by a very large pressure-related response. Our observations demonstrate that blood pressure fluctuations can exert confounding effects on brain NIRS, through expression in extracranial tissues and within the brain itself. We highlight the necessity for continuous blood pressure monitoring alongside brain NIRS, and for further research on methods to correct for physiological confounds.

## Highlights

► We recorded NIRS from the visual cortex during pattern-reversal stimulation. ► Transient blood pressure changes were induced by arm-raising. ► Blood pressure changes significantly altered deep and shallow NIRS recordings. ► The effect appears to originate from both intra- and extra-cranial regions. ► Blood pressure must be considered as a potential confound in NIRS studies.

## 1 Introduction

Near-infrared spectroscopy (NIRS) of the brain is receiving increasing interest as a functional neuroscientific technique, complementing neuroelectric approaches such as electroencephalography (EEG) and high-cost neuroimaging using functional magnetic resonance imaging (fMRI) or positron emission tomography (PET).

Like fMRI, brain NIRS is typically used to measure haemodynamic changes related to cortical neural activity. NIRS devices emit one or more incident beams via transmitting optodes through the scalp and skull. The light is back-scattered and follows a ‘banana-shaped’ wavepath through superficial cortical regions towards the receiving optodes. Using appropriate wavelengths, it is possible to quantify changes in the concentrations of oxy- and deoxyhaemoglobin, which have distinct absorption spectra. While brain NIRS shares a common physiological basis to fMRI, it offers several potential advantages in terms of logistics (cost, equipment portability, patient compatibility) and data quality (temporal sampling density >10 Hz vs. <1 Hz). In contrast with EEG, the source localization problem is not ill-posed, as the recording regions are straightforwardly determined by the optode positions. A variety of NIRS devices are available, ranging from inexpensive wearable implementations based on light-emitting diodes and photodiodes, to large multichannel systems employing pulsed laser sources and photomultiplier detectors (Strangman et al., 2002; Wolf et al., 2007; Lloyd-Fox et al., 2010).Brain NIRS has established clinical application for monitoring brain oxygenation in neonatological and intensive-care settings. As a research tool, there is growing evidence for its utility across a range of active experimental paradigms probing motor and language function, executive control and appraisal of affective stimuli (Strangman et al., 2002; Wolf et al., 2007; Lloyd-Fox et al., 2010). Nevertheless, these cognitive and emotional processes are also known to affect peripheral physiology, influencing heart rate, respiration, blood pressure and skin perspiration (Jänig et al., 2006; Critchley, 2009). In particular, transient blood-pressure increases are frequently observed, e.g., in response to positive or negative emotions, mental effort and response conflict (Hanson et al., 1993; Minati et al., 2009).

In brain NIRS, the incident and backscattered beams cross skin, subcutaneous fat and scalp muscles, which are heavily vascularised. As a consequence, measurements are sensitive to haemodynamic changes occurring in these extracerebral tissues. Within the brain, haemodynamic parameters are closely regulated through tight neurovascular coupling and partial autonomic control (Zhang et al., 2002). However, outside the brain, regional and systemic changes in cardiovascular state strongly influence the local concentration of oxy- and deoxyhaemoglobin (Nishiyasu et al., 1999; Hachiya et al., 2008). Thus, autonomically mediated peripheral cardiovascular responses can confound NIRS haemodynamic indices of neural activity if not taken into account.

Coupling is observed between blood pressure and the haemodynamic responses measured using NIRS. For example, in one recent study, NIRS was used to record visually evoked haemodynamic responses during the processing of affective pictures carrying distinct valences: the emotional content modulated response amplitudes measured in the early visual areas but, in parallel, engendered changes in systemic blood pressure. These two effects were significantly correlated such that entering mean arterial pressure (MAP) as a nuisance covariate in the NIRS analysis reduced the significance of differences between picture types (Minati et al., 2009). Another study used NIRS to record haemodynamic responses over prefrontal cortex during anagram solving, and reported that task difficulty modulated both the measured haemodynamic response and MAP. In some participants, the association between the two responses was significant at the individual level (Tachtsidis et al., 2008), and the NIRS responses to cortical haemodynamic vs. blood pressure changes were indistinguishable overall (Tachtsidis et al., 2009). The clinical literature similarly acknowledges correlations between continuously recorded brain NIRS signals and MAP fluctuations in paediatric, neurosurgical and cardiac patients (Yoshitani et al., 2007; Weerakkody et al., 2010).

These observations of correlations do not necessarily imply causative effects of transient blood pressure changes on the recorded brain NIRS responses. Systemic and cortical haemodynamic effects may be highly correlated because they share a common effector, for example activation of the amygdala by emotionally valenced stimuli may trigger systemic autonomic cardiovascular responses while also enhancing activity within early visual cortical regions (Vuilleumier et al., 2002; Critchley et al., 2005). The two mechanisms are not mutually exclusive.

It is well established that in patients with autonomic failure postural changes such as standing up or the table tilt-test produce cerebral oxygenation changes which are strongly coupled with systemic blood pressure. In healthy controls, the association between the two variables is not as strong (Harms et al., 2000; Tachtsidis et al., 2004; Hunt et al., 2006). It remains to be determined whether the much smaller and relatively shorter systemic blood pressure changes engendered by performance of a task can have a significant confounding effect on brain NIRS, and quantify the magnitude of this effect. Addressing this issue, we performed two independent manipulations while recording from visual cortex using methods established in an earlier study (Minati et al., 2009). Cortical haemodynamic responses were induced through visual stimulation using reversing checkerboards of varying duration. Simultaneously, transient blood pressure changes independent of visual stimulation were generated by rapid arm-raising.

## 2 Methods

### 2.1 Participants

Sixteen participants (9 F, 7 M), age 25 ± 7 (range 19–42) years were enrolled after providing written informed consent. The study was approved by the Brighton and Sussex Medical School Research Governance and Ethics Committee (BSMS-RGEC). All participants had normal or corrected-to-normal vision, and all but one were right handed. None was taking psychoactive or cardiovascular medication. A ‘deep-penetration’ optode configuration was used to record data from nine participants and a ‘shallow-penetration’ optode configuration from the remaining seven (see below).

### 2.2 Experimental procedure

The study was conducted with participants seated in the psychophysiological laboratory. Ambient lighting was darkened after positioning the NIRS optodes, and data were recorded in two 15 min-long sessions (alternating the side on which blood pressure was measured with the side on which the arm was lifted).

Participants fixated on the centre of visual stimuli consisting of radial black/white chequerboards reversing at a rate of 5 Hz and presented for either 1500 or 3000 ms. The centre was marked by a red cross, which persisted between stimuli. The surrounding chequerboard occupied 80% of a 19 in. CRT screen positioned 1 m away from the head. The resting condition consisted of fixating the cross superimposed to a black screen.

Transient blood pressure increases were induced through a motor act, i.e. by laterally raising a straight arm (until elbow at level of the eyes, hand reaching upwards as high as possible) while the rest of the body remained still. This action was performed as rapidly as possible with gaze remaining fixated on the central. For half the visual stimuli, 1 s prior to onset an upwards arrow was presented for 500 ms to prompt arm lifting (generally completed in less than 1 s). Twelve seconds after visual stimulus onset, a downwards arrow signalled to lower the arm slowly, until completely relaxed with the hand hanging towards the floor. For the other half of the visual stimuli (no-movement trials), horizontal arrows were displayed for visual matching. Participants practiced the task, i.e. rapid arm-lifting with minimal movement of the rest of the body, several times prior to data acquisition. They were observed throughout the whole session by the experimenter, who ensured that movement of the head and contralateral arm was minimized.

Each stimulus was presented 15 times, yielding a total of 60 stimuli which were delivered in randomized order. The inter-trial interval was jittered, with 19.2 ± 2.9 s.

### 2.3 Data acquisition

NIRS measurements were acquired by means of a continuous wave device emitting at 764 ± 20 nm and 859 ± 20 nm, and recording at 50 Hz (Oxymon Mk III, Artinis BV, Zetten, NL). Four channels were used, positioning two receiver optodes above and below the Oz site (as defined by the 10/20 system), and two transmitter optodes left and right of Oz.

For the 9 participants from whom data were recorded in deep-penetration mode, the transmitter–receiver distance was 4 cm. This was implemented positioning the optodes approximately 2.8 cm away from Oz. The resulting ‘banana-shaped’ wavepath, penetrating a depth of about 2 cm, traversed a combination of striate and extrastriate regions, principally lying within the expected localization of visual areas V1 and V2 (Al-Rawi et al., 2001; Minati et al., 2009; Mansouri et al., 2010). When recordings were made in shallow-penetration mode (7 participants), the inter-optode distance was 1 cm, implemented by positioning the optodes 7 mm away from Oz. The wavepath penetrated by about 5 mm, traversing scalp, subcutaneous fat and muscles without fully entering the intracranial compartment (Al-Rawi et al., 2001; Mansouri et al., 2010).

The optodes holder consisted of a tightly fitting modified silicone swimming cap (thickness ∼1 mm, super-stretch type), to which a nylon screw insulator and two nylon washers (RS no. 178-800 and 280-521) had been secured corresponding to the intended optode positions. The optodes were firmly held in place by a 5 cm-thick sponge secured with Velcro straps. In order to avoid weight loading, the optical fibres were supported by a flexible metal arm positioned placed close to the back of the head. The fibres did not touch the body to minimize the possibility of optode movement.

Changes in oxy- (O_2_Hb) and deoxyhaemoglobin (HHb) concentration were calculated through the modified Beer–Lambert law, including the differential path length factor as given by the age-dependence formula introduced by Duncan and co-workers (Duncan et al., 1996; Strangman et al., 2002; Wolf et al., 2007; Lloyd-Fox et al., 2010).

Beat-to-beat arterial pressure was recorded through volume-clamping of the finger pulse, as implemented by the Finometer device (Finapres Medical Systems BV, Arnhem, The Netherlands), on the side contralateral to the arm-movement. A height reference sensor was used to remove confounds related to arm position. Calibrations were synchronized with stimulus presentation through an RS232 serial link, and programmed to occur automatically before each trial.

### 2.4 Data analysis

All raw NIRS traces were visually inspected to confirm the absence of transient changes synchronous with arm-movement, which would suggest motion contamination.

Recorded NIRS signals were low-pass filtered at 1 Hz, then detrended by fitting the baseline with a 5th degree polynomial which was subsequently subtracted. The resulting time-courses were epoched between −0.5 and 12 s with respect to visual stimulus onset, and the average pre-stimulus (0.5 s) baseline was subtracted. After rejection of outlier epochs (using a ±3 SD criterion), the remaining data were averaged according to the two experimental factors. Haemodynamic response amplitude and latency (defined, respectively, as the maximum/minimum amplitude over pre-stimulus baseline and the associated time with respect to stimulus onset) were thereafter determined.

For blood pressure, the volume clamping pressure signal was low-pass filtered at 10 Hz and pre-processed by means of a peak-picking algorithm to yield time-courses for systolic, diastolic and mean arterial pressure. As above, these were detrended polynomially, then epoched between −0.5 and 10 s to compute, for each trial, response amplitude and latency as above.

One sample *t*-tests with respect to zero were performed to confirm whether arm-raising induced significant changes in systolic, diastolic and mean arterial pressure. Linear regression was used to determine correlations between systolic and diastolic pressure. O_2_Hb and HHb responses following arm-raising recorded in shallow-penetration mode were similarly evaluated using one-sample *t*-tests, and linear regressions were performed between these signals and MAP.

For the deep-penetration measurements, three planned comparisons were considered, using two-sample *t*-tests separately for response amplitude and latency: (1) contrasting 1500 ms- with 3000 ms-persistency visual stimuli in the absence of arm-raising, (2) contrasting the same for arm-raised trials and (3) contrasting all arm-raised vs. all no-movement trials. Correcting for multiple comparisons, we set *p*_CRIT_ = 0.05/3 = 0.017 for these tests. As above, linear regressions were performed to test for correlations between the O_2_Hb and HHb responses and MAP, separately for arm-raised and no-movement trials.

## 3 Results

**Fig. 1:**
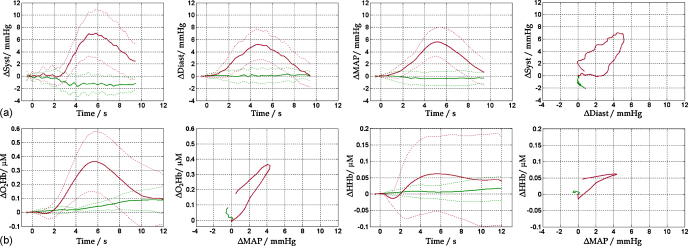
(a) Transient blood-pressure increase induced by arm-raising: time-courses of systolic, diastolic and mean arterial pressure (MAP), and correlation between systolic and diastolic pressure changes. (b) Corresponding effect on O_2_Hb and HHb signals measured in ‘shallow’ optode configuration, and correlation with MAP. Solid lines represent the average of arm-raised (redviolet) and no movement trials (bluegreen). Dashed lines denote 1 SD. (For interpretation of the references to colour in this figure legend, the reader is referred to the web version of this article.)

Arm-raising produced a transient increase in systolic blood pressure over pre-stimulus level (mean ± SD across participants; 7.8 ± 4.1 mmHg; *t*(15) = 7.7, *p* < 0.001), peaking at 5.8 ± 0.9 s (Fig. 1a). Similarly, increases were evident in diastolic pressure (5.6 ± 2.4 mmHg; *t*(15) = 9.4, *p* < 0.001; peaking at 5.1 ± 0.8 s) and MAP (5.9 ± 2.5 mmHg; *t*(15) = 9.4, *p* < 0.001; peaking at 5.3 ± 0.8 s). Systolic and diastolic pressure changes were significantly correlated (*r* = 0.7, *p* < 0.001), yet diastolic pressure returned to baseline more rapidly.

Upon visual inspection of all raw NIRS traces, no transient change suggestive of movement contamination was found corresponding to any arm-movement act.

In shallow-penetration NIRS recordings from the occipital region, arm-raising produced a transient increase in O_2_Hb signal (0.39 ± 0.24 μM over pre-stimulus level; *t*(6) = 4.7, *p* = 0.003; peaking at 6.0 ± 0.9 s; Fig. 1b). Arm-raising also increased average HHb signal (0.08 ± 0.14 μM, peaking at 5.9 ± 3.0 s) but this effect did not reach the significance threshold. A significant positive correlation with MAP was observed for both O_2_Hb and HHb signals during arm-raised trials (*r* = 0.6, *p* < 0.001).

**Fig. 2:**
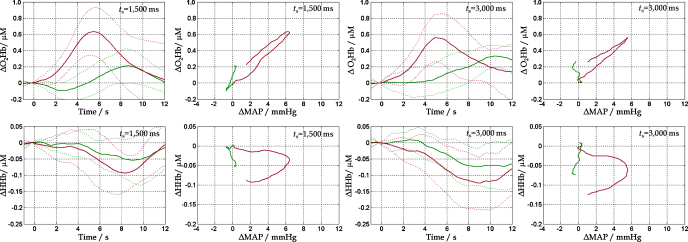
Evoked O_2_Hb and HHb responses during visual stimulation for 1500 ms and 3000 ms, measured in ‘deep’ optode configuration, and correlation with MAP. Solid lines represent the average of arm-raised (redviolet) and no movement trials (bluegreen). Dashed lines denote 1 SD. (For interpretation of the references to colour in this figure legend, the reader is referred to the web version of this article.)

In deep-penetration recordings, both visual stimulation and arm-rising generated positive O_2_Hb and negative HHb responses (Fig. 2). In the absence of arm-raising, the O_2_Hb response occurred later for the 3000 ms compared to the 1500 ms visual stimuli (10.4 ± 1.7 s vs. 8.6 ± 1.0 s, *t*(8) = 3, *p* = 0.01). Arm-raising abolished this differential effect (6.9 ± 2.9 s vs. 6.4 ± 2.2 s, *p* = 0.7), and markedly reduced latency (6.7 ± 2.3 s vs. 9.5 ± 1.3 s, *t*(8) = 3.7, *p* = 0.006). The O_2_Hb response amplitude was not sensitive to the duration of visual stimulation (0.38 ± 0.16 μM vs. 0.26 ± 0.18 μM, *p* = 0.2), but it was significantly larger for arm-raised trials (0.69 ± 0.25 μM vs. 0.32 ± 0.15 μM, *t*(8) = 4.4, *p* = 0.002). In the absence of arm-raising, the HHb response latency was not sensitive to the duration of visual stimulation (8.8 ± 4.0 s vs. 7.4 ± 3.8 s, *p* = 0.5), and there was no additional effect of arm-raising (8.6 ± 1.4 s vs. 8.1 ± 2.7 s, *p* = 0.6). The HHb response amplitude was not sensitive to the duration of visual stimulation (−0.11 ± 0.05 μM vs. −0.09 ± 0.04 μM, *p* = 0.3), however there was a borderline effect of arm-raising, which did not survive correction for multiple comparisons (−0.15 ± 0.06 μM vs. −0.08 ± 0.03 μM, *t*(8) = 2.3, *p* = 0.04).

For arm-raised trials, there was a strongly significant positive correlation between O_2_Hb response and MAP (*r* = 0.93, *p* < 0.001 for 1500 ms and *r* = 0.95, *p* < 0.001 for 3000 ms visual stimuli). There was a looser correspondence between HHb and MAP, reflected in different slopes of the rising and falling parts of the response (Fig. 2), with a nevertheless significant negative correlation (*r* = 0.19, *p* = 0.003 for 1500 ms and *r* = 0.29, *p* < 0.001 for 3000 ms stimuli).

## 4 Discussion

The key findings of this study are that (1) transient systemic blood pressure changes are reflected in brain NIRS measurements, and (2) the coupling appears to take place in both extracranial tissues and the brain itself, with different patterns. These results signal the need to monitor and correct for blood pressure effects when brain NIRS is used to investigate cortical haemodynamics.

Arm-raising generated transient increases in systemic blood pressure. There are a number of possible mechanisms for this including the central command driving local and systemic facilitatory cardiovascular responses in anticipation and in response to effort (Smith et al., 2000), and the sudden shift between two hydrostatic states, i.e. when the arm is lowered all its blood volume is below the level of the shoulders whereas the opposite holds when it is raised. The magnitude of blood pressure responses to arm-raising (∼6 mmHg) was larger than those typically evoked by individual visual stimuli (∼2 mmHg; e.g., Minati et al., 2009), and closer to values expected for extended blocks of demanding task performance (∼5 mmHg; e.g., Tachtsidis et al., 2008). The response time was, however, similar to that of a typical haemodynamic response evoked by a short stimulus, confirming the suitability of this manipulation for studying the effect of blood pressure changes during an event-related task.

Shallow recordings show that arm-raising evokes increases in O_2_Hb concentration which significantly correlate with MAP. These clearly demonstrate that transient blood pressure manipulation affects the oxygenation and perfusion of subcutaneous extracranial tissues. Since O_2_Hb was increased in the absence of a corresponding HHb decrease, the observed effect most likely reflects enlarged blood volume, consequential to vascular elasticity, rather than increased blood flow (Nishiyasu et al., 1999; Strangman et al., 2002; Hachiya et al., 2008).

In the absence of blood pressure changes induced by arm-raising, deep (i.e. cortical) NIRS recordings showed increases in O_2_Hb and decreases in HHb following pattern-reversal chequerboard stimulation consistent with neurally coupled haemodynamic responses. The latency of the O_2_Hb response was correspondingly sensitive to stimulus duration. However, blood pressure manipulation by arm-raising induced a positive O_2_Hb response, which was more than twice as large as that obtained through visual stimulation alone. Here, the correlation between the O_2_Hb response and MAP was even stronger than for shallow recordings, implying that the coupling was also present intra-cranially. This is in line with literature on autonomic control of brain vasculature (Zhang et al., 2002). The finding of a weak negative HHb response, however, suggests that a different mechanism may be at play in the brain, i.e. that increased blood pressure elevates blood flow, leading to increased O_2_Hb concentration and dilution of HHb, as commonly observed for cortical haemodynamic responses (Strangman et al., 2002). This decoupling is expected, considering that regional blood volume is more tightly and rapidly regulated in the brain than in the muscles (Zhang et al., 2002; Banaji et al., 2008). Accordingly, the correlation between the HHb response and MAP was weaker than that observed for shallow recordings.

In arm-raising trials, the response latency difference observed between 1500 and 3000 ms visual stimuli was not detectable and interestingly, the stimulus-evoked O_2_Hb responses were flattened, suggesting that increased systemic MAP does not additively enhance neurally driven haemodynamic activation. Our observations are in line with empirical data regarding non-linearity and time-dependence of cerebrovascular responses and support the notion of multiple interacting systems involved in the control of regional cerebral perfusion (Banaji et al., 2008).

This study has several limitations that need to be considered. First, as with most other NIRS studies the wavepath and penetration depth were assumed a priori, on the basis of existing literature. More solid indication of the attained penetration depth would need to be obtained using Monte Carlo photon diffusion simulations, based on the segmentation of the individual structural scans (e.g., Mansouri et al., 2010). Second, the number of participants was relatively small, especially for the shallow-recordings group; as a consequence, the findings should be interpreted as preliminary. Third, we are unable to directly confirm whether the source of the effect is wholly extra-cranial, or whether an intra-cranial effect is present as well. To this end, the experiment needs to be replicated using time-resolved spectroscopy, which enables one to gate the measurements to a specific penetration depth range (Torricelli et al., 2008).

In spite of these limitations, these results extend previous correlational findings by unequivocally demonstrating that blood pressure changes can exert strong confounding effects on brain NIRS measurements. Our findings have specific relevance to the implementation of NIRS as a functional neuroscience method. Moreover, since autonomically mediated neurovascular effects are also present within the brain itself (Zhang et al., 2002), they may also have more general relevance to other techniques including fMRI that depend on neurally coupled haemodynamic responses evoked by stimuli or responses that may also generate systemic effects. There are, in fact, a number of signal processing methodologies that can be used to address the issue of systemic confounds. One approach, discussed by Tachtsidis et al. (2010), is to measure and explicitly model the physiological confounds, inserting them as nuisance regressors. An alternative, discussed in Katura et al. (2008), is to use separation and classification techniques such as independent component analysis (ICA) and *k*-means clustering to isolate task-related from physiology-related activity components, purely on the basis of the fNIRS or fMRI time series.

More work is needed to explore the effect of smaller blood pressure manipulations and to characterize the response function mapping different magnitudes of blood pressure change to their effects on O_2_Hb and HHb concentrations. This will enable inclusion of blood pressure-derived measurements as nuisance covariate in statistical analyses. Our findings do not definitively clarify the relative contribution of extra- vs. intra-cranial coupling, and the experiment needs to be replicated with time-resolved spectroscopy. Relevant confirmation of our findings should also be obtained simultaneously recording from deep- and shallow-separation optodes, through a NIRS system with a larger number of channels. Further investigations are necessary to clarify to what extent MAP changes may affect tasks structured in longer blocks rather than individual events. Even before such data becomes available, in light of our observations blood pressure effects should always be characterized during functional NIRS experiments.

## Conflicts of interest

All authors declare that they do not have any real or perceived conflicts of interest pertaining to the present study.
